# Seizure Prediction and Detection via Phase and Amplitude Lock Values

**DOI:** 10.3389/fnhum.2016.00080

**Published:** 2016-03-08

**Authors:** Mark H. Myers, Akshay Padmanabha, Gahangir Hossain, Amy L. de Jongh Curry, Charles D. Blaha

**Affiliations:** ^1^Department of Anatomy and Neurobiology, University of Tennessee Health Science CenterMemphis, TN, USA; ^2^Department of Electrical and Computer Science, Massachusetts Institute of TechnologyBoston, MA, USA; ^3^Department of Electrical Engineering and Computer Science, Texas A&M UniversityKingsville, TX, USA; ^4^Department of Biomedical Engineering, University of MemphisMemphis, TN, USA; ^5^Department of Psychology, University of MemphisMemphis, TN, USA

**Keywords:** patient-based, phase-amplitude lock value, seizure prediction, seizure detection, Hilbert transform

## Abstract

A robust seizure prediction methodology would enable a “closed-loop” system that would only activate as impending seizure activity is detected. Such a system would eliminate ongoing stimulation to the brain, thereby eliminating such side effects as coughing, hoarseness, voice alteration, and paresthesias (Murphy et al., 1998; Ben-Menachem, 2001), while preserving overall battery life of the system. The seizure prediction and detection algorithm uses Phase/Amplitude Lock Values (PLV/ALV) which calculate the difference of phase and amplitude between electroencephalogram (EEG) electrodes local and remote to the epileptic event. PLV is used as the seizure prediction marker and signifies the emergence of abnormal neuronal activations through local neuron populations. PLV/ALVs are used as seizure detection markers to demarcate the seizure event, or when the local seizure event has propagated throughout the brain turning into a grand-mal event. We verify the performance of this methodology against the “CHB-MIT Scalp EEG Database” which features seizure attributes for testing. Through this testing, we can demonstrate a high degree of sensivity and precision of our methodology between pre-ictal and ictal events.

## Introduction

Seizure prediction based on electroencephalograms (EEG)/intracranial EEG (iEEG) is complicated by two factors. The first is that preictal and interictal EEG/iEEG patterns across patients vary substantially. There may be no single generic algorithm that can be applied to all patients and can achieve high sensitivity (Osorio et al., 1998; Shoeb et al., 2009). The second is that EEG/iEEG is highly complex and varies over time, and no single measure of EEG/iEEG has yet been predictive on its own (Mormann et al., 2005, 2007). Therefore, we hypothesize that a patient-specific classification method based on multiple features extracted from EEG/iEEG will achieve high sensitivity. Seizure detection analysis includes the testing of pre-ictal (pre-seizure) and seizure occurrences. The robustness of any seizure prediction algorithm must also take into consideration inter-ictal events in order to test false detection occurrences through instances where there are no impending seizures.

Park et al. (2011) propose a patient-specific algorithm for seizure prediction using multiple features of spectral power from EEG and support vector machine (SVM) classification. Their patient-specific algorithm for seizure prediction has a sensitivity of 97.5% with total 80 seizure events. Their prediction rate demonstrates that seizures can be predicted by the patient-specific approach using linear features of spectral power and nonlinear classifiers. Gamma frequency bands were the most discriminating in eight patients, indicating that time differential preprocessing may reveal spectral changes more indicative of a preictal event. Time-differential processing using the Hjorth mobility parameter (Hjorth, 1970) normalizes the power in each band by measuring its contribution to the total power by flattening the spectrum, making power in the high frequency bands similar to that in low frequency bands.

Mormann et al. (2000) reported changes in phase synchronization between different brain areas before seizure occurrences. In two reviews of their own work, Le Van Quyen et al. (2001, 2003) referred to a submitted study including eight patients with neocortical epilepsy that seemed to confirm these findings. Chavez et al. (2003) published results using phase synchronization analysis after band-pass filtering of the EEG and reported pre-ictal changes in synchronization to occur predominantly in the beta band. A better performance was reported for bi- and multi-variate measures (Iasemidis et al., 2005; Le Van Quyen et al., 2005) whereas Mormann et al. (2005) observed pre-ictal changes to be locally restricted to specific channels rather than occurring as a global phenomenon. Their pre-ictal period was between 4–221 min. According to their review of seizure detection algorithms, Mormann et al. (2007), state that if prediction algorithms are optimized using training data, i.e., pre-ictal data, they should be tested on interictal data.

According to Snyder et al. (2008), seizure prediction algorithms must perform better than chance. Investigators address this challenge through a variety of approaches: comparisons between pre-ictal and interictal states, surrogate data sets, periodic predictors, and random or pseudo-random processes (Andrzejak et al., 2003; Chaovalitwongse et al., 2005). In Snyder’s article, they consider a seizure advisory algorithm that processes EEG data and produces a binary data classification of: 1 if preictal, 0 if interictal. Their approach follows the same interval classification as Winterhalder et al. (2003) and Schelter et al. (2006), where the preictal interval corresponds to the seizure prediction horizon (SPH), but they define the seizure occurrence period (SOP) as the sum of SPH’s as opposed to the ictal period. The seizure prediction algorithm involves spectral power feature extraction and patient-specific classification. Their algorithm analyzed all available channels of cortical potentials including electrodes placed on and surrounding the seizure focus, as well as a reference electrode well-separated from the focus. The EEG signal was band-pass filtered to beta band (16–32 Hz) using a FIR filter design, with a 5 s sliding window scheme displaced in 1 s increments. Using kNN’s for feature classification, their seizure prediction sensivity for four patients were between 60–100%. One of the key aspects of this study discusses the performance between seizure and chance prediction distributions. Their study highlights the idea how far in advance of seizures the epileptic network becomes “proictal” (i.e., enters a state with a high probability of seizure onset), as opposed to the algorithm evaluator predefining rigid preictal vs. non-preictal time windows.

In a study by Cook et al. (2013), 11 patients were implanted with a seizure prediction device, with a performance estimate sensitivities ranging from 65 to 100%. Their seizure detection algorithm was based on an unsupervised learning approach that identifies significant outliers in features of EEGs that are associated with seizures (Gardner et al., 2006). Only electrographic events that were judged by a reviewer to be associated with clinical manifestations (seizure diary or audio recordings; i.e., clinically correlated seizures) or those that were electrographically similar in onset, propagation, and spread to clinically correlated seizures (i.e., clinical equivalent seizures) were used to train the algorithm (Cook et al., 2013). False negatives were measured as preceding seizure activity vs. randomized activity, and pre-seizure activity had to precede a seizure by 5 min in order for it to be deemed a true-positive. A predicted seizure which did not occur within 5 min of a seizure occurrence would qualify as a true-negative. Their study involves a stringent criterion for pre-seizure activity which analyzed seizure activity within a fixed period before the SOP.

Mathematical analysis of the spatiotemporal dynamics found in EEG recordings of patients with medically intractable epilepsy have discovered a preictal transition that precedes seizures for periods on the order of minutes to hours (Niedermeyer, 1987; Skarda and Freeman, 1987; Martinerie et al., 1998; Schachter and Saper, 1998; Sackellares et al., 1999, 2000; Tsakalis et al., 2005; Navarro et al., 2007). Our approach consists of locating the fast changes of phase and amplitude of the signal, and therefore can provide a finely tuned method that finds seizures lasting seconds and interictal markers lasting 1–2 s as opposed to studies that involve longer term seizure states.

Seizure activity is characterized by recurrent, short-term electrical discharges of the cerebral cortex that result in intermittent disturbances of brain function. The state between seizures, known as interictal behavior, appears to have minor spiking activity. In seizures of focal onset (e.g., focal seizures and partial seizures), the anatomical distribution of the interictal spikes varies, but spikes tend to occur most commonly in the epileptogenic zone and its connections. During the seizure, organized, semi-periodic electrical discharges develop in the epileptogenic zone and spread, within seconds, over widespread areas of cerebral cortex. Seizure discharges typically last seconds to minutes and are followed by the normal non-linear, chaotic neuron firing behavior that is captured through an EEG. Through mathematical analysis of the spatiotemporal dynamics found in EEG recordings of patients with medically intractable epilepsy, researchers have discovered a preictal transition that precedes seizures for periods on the order of minutes to hours (Le Van Quyen et al., 2005). This preictal dynamical transition is characterized by a progressive convergence (entrainment) of dynamical measures at specific anatomical areas in the neocortex and hippocampus. Here, we have focused on looking for quantifiable spatial and temporal shifts in preictal synchronization far in advance of seizure onset detectable on the EEG. Because normal EEG is enormously varied, manifesting qualitative changes depending on behavioral state, it is important to clearly distinguish preictal changes from all the other interictal states. We have confirmed that there are prediction markers found within the interictal state that presage the eventual seizure state.

In the presence of random chance that two or more neural populations would synchronize, there are two fundamental conditions for these phenomena to occur. There must be some degree of coupling between the neural populations, and there must exist at the very least some semi-periodic neural behavior within one of the coupled neural populations. Therefore the measurement of phase locking due to pre-seizure and ongoing seizure behavior must involve an EEG time series from an epilepsy patient for the prediction and detection algorithm to be relevant. The following sections demonstrate the underlying mechanisms of neural synchronization which separates random synchronization to entrained synchronized neurological populations.

### Network of Coupled Neural Oscillators

Phase synchronization or “locking” between neural populations does not occur by random chance. Phase synchronization occurs in the presence of the entrainment of neural populations. In order to better understand this phenomena, theoretical studies of phase synchronization of chaotic oscillators (Rosenblum et al., 1996), can be applied in neuroscience where synchronization processes are of crucial importance, e.g., for neuro-pathologies such as epilepsy. Synchronization of two periodic non-identical oscillators is understood as adjustment of their rhythms, or appearance of *phase locking*, due to interaction. Phase locking is equivalent to the concept of frequency locking, Ω_1_–Ω_2_, where Ω_1,2_ = <ϕ_1,2_>, and brackets mean time averaging. ϕ_1,2_ are phases of two oscillators, and ϕ_n,m_ is the phase difference, where ϕ_n,m_(t) = ϕ_1_(t) ϕ_2_(t). All phases are divided by 2π for normalization, and ϕ_n,m_ are not defined on the circle [0, 1] but on the whole real line (Tass et al., 1998). The phase component of the signal is calculated via the arctan(imaginary/real) part of the signal via Hilbert transformations (Freeman, 1986).

Phase synchronizations are governed by the amount of noise within the oscillators, and the coupling strength between the oscillators. With noise and a relatively small coupling strength, the oscillators approach unlocked orbits over a time series. As coupling between the chaotic oscillators increase, phase synchronizations form rapidly and de-synchronize over time. If stronger noise is added, phase slips occur more frequently, and synchronization happens less often over the same length of time. Therefore, neural population’s exhibit a degree of noise that maintains independent basal activity, yet neural population synchronization can occur if a dominant waveform form emerges from a local neural area (Kozma and Freeman, 2003; Kozma et al., 2007; Myers and Kozma, 2007). If there exists a strong coupling between neural populations, phase synchronization will propagate throughout the network, as seen in focal seizures that emerge into grand-mal seizures.

## Materials and Methods

### Measuring the Degree of Synchrony Between Channels: Seizure Prediction

**Figure 1:**
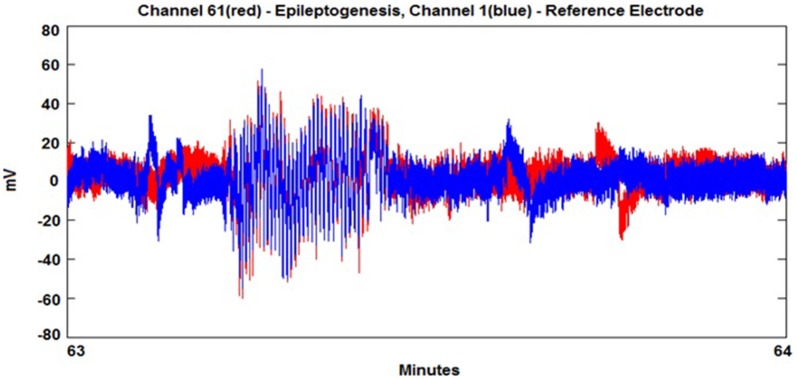
**A seizure event where working electrode (red) entrains with reference electrode (blue)**.

In order to avoid spurious detection of locking due to noise and small oscillation coupling, we initially band pass filter the time series in order to focus on the frequency areas that produce pre-ictal and ictal behavior. Seizure prediction analysis begins with the decomposition of EEG via Hilbert transformation. The signal is split into two parts, the analytic amplitude (AA) and the analytic phase (AP). The AA and AP are utilized in equations 1 and 2 in order to measure the level of phase and amplitude synchrony between EEG signals from paired electrodes using a sliding window of *n* = 1000, 2500, or 5000 data points (Niedermeyer, 1987). The sliding window acts as a filtering method to sum a larger group of points to calculate Phase Lock Value (PLV). These synchrony levels are termed PLV and Amplitude Lock Value (ALV) and are determined using electrodes in the areas of the scalp where seizure behavior (working electrode) and “normal” EEG behavior (reference electrode) is found, see Figure 1. The PLV is calculated in the following manner:

$$\text{PLV}=\left\|\frac{1}{\text{n}}\sum_{1}^{\text{n}}{\text{e}}^{\text{i}\left[\phi_{1}(\text{t})\;-\;\phi_{2}(\text{t})\right]}\right\|$$

where ϕ is the analytic phase (AP). The value “*n*” refers to the window size to sum PLV values, and “t” refers to the phase at time “t” within the time series. PLV varies between independent signals and constant phase-lag between the two signals, i.e., EEG signals will either synchronize or operate independently (Le Van Quyen et al., 2005). The analytic amplitude (A) is used to measure the synchrony between two channel’s amplitudes or the ALV:

$$\text{ALV=}\left\|\frac{1}{\text{n}}\sum_{1}^{\text{n}}{\text{e}}^{\text{i}\left[A_{1}(\text{t})-A_{2}(\text{t})\right]}\right\|$$

The ALV measurement is used in conjunction with the PLV to identify the seizure state. The degree of amplitude locking between two channels determines the similarity between the two amplitudes.

**Figure 2:**
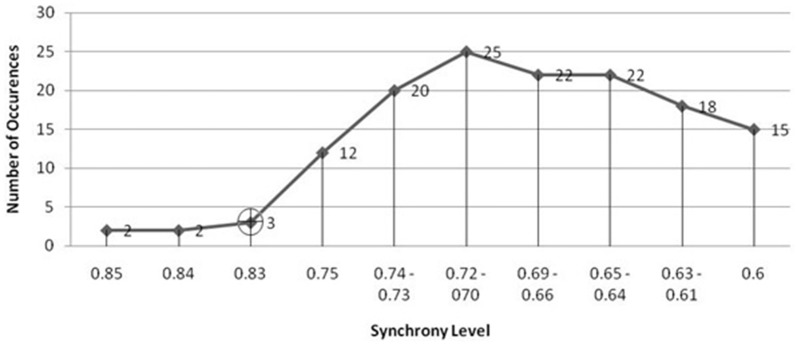
**Threshold selection is based on finding the lowest number of Phase Lock Value (PLV) values just before the amount of values rise dramatically.** The PLV begins to rise at the synchrony level “0.83” (highlighted by the black cross) which establishes the threshold marker on the PLV display. This figure demonstrates that the smallest number of PLV values at a synchrony level range, i.e., between 0.6 and 0.9 correspond to those values that are higher than the rest of the PLV values that correspond to non-synchronous channel pairs. These higher PLV values correspond to pair-wise channel synchronization. The threshold value “0.83” is selected in order to separate “normal” chaotic neural activity from highly synchronized neural activity found in the seizure state. As the slope of the number of PLV occurrences vs. synchrony level rises sharply from “0.83” and “0.75”, we can determine the threshold value between normal and seizure activity for this patient’s EEG activity.

**Figure 3:**
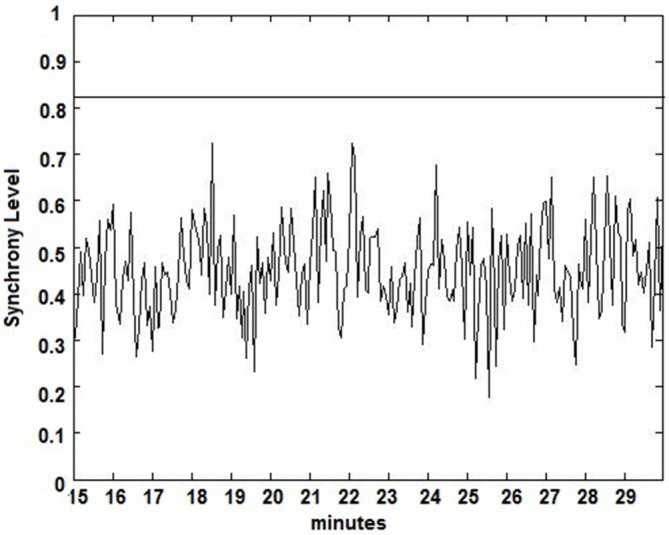
**Calculated PLV over an interictal time series.** Calculated PLV values remain below the patient-specific threshold calculated from Figure 2.

**Figure 4:**
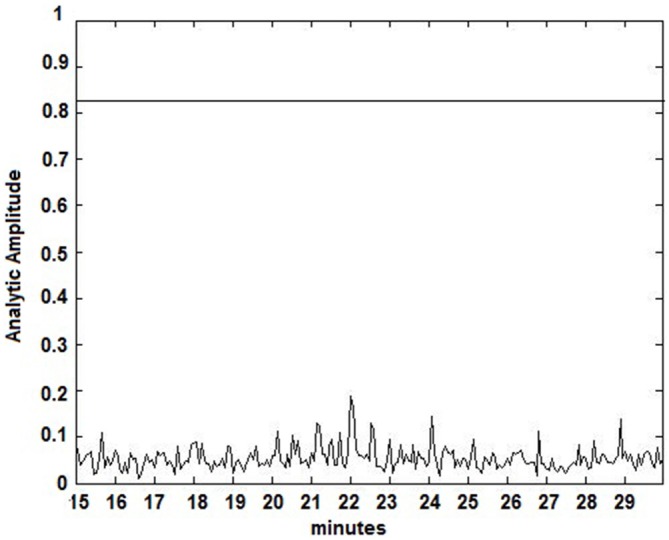
**Amplitude Lock Values (ALV) over an interictal time series.** Calculated ALV values remain below the patient-specific threshold calculated from Figure 2.

**Figure 5:**
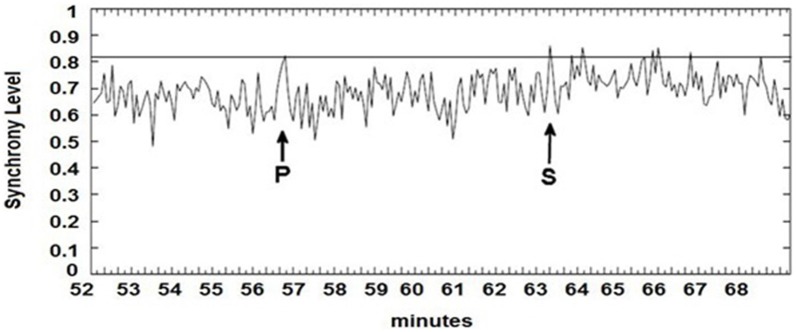
**Calculated PLV over pre-ictal time series.** Prediction marker (P) and seizure event (S) are signified by arrows. Prediction markers and seizure events are found by the rise of PLV values above a patient-based threshold.

**Figure 6:**
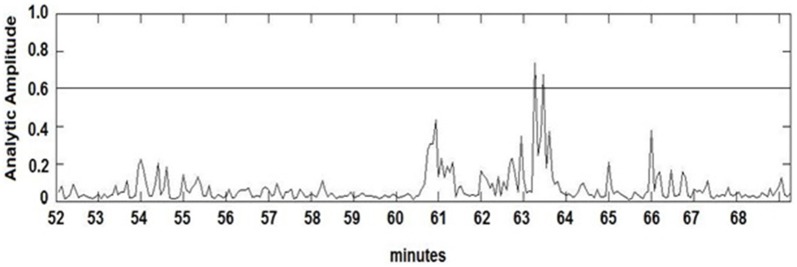
**Amplitude Lock Values (ALV) over pre-ictal time series.** Seizure events are found by the rise of ALV values above a patient-based threshold, which also correspond to the rise of PLV values.

The seizure prediction and detection methodology is as follows. PLV and ALV are calculated between the working and reference electrodes. A threshold value of the PLV and ALV is used as a detection marker to indicate seizure occurrence. The threshold value of the PLV alone is used as a prediction marker. The optimal PLV and ALV thresholds are determined retrospectively for each patient. For an accurate prediction, the prediction marker must be set within an appropriate time before a seizure occurrence. Initially, a time interval after the prediction marker occurs is set, which is called the SPH (Spencer et al., 1992). The end of the time interval of the SPH is the SOP. Our algorithm constitutes a SPH + SOP prediction horizon. During interictal periods, (i.e., periods far away from any seizure), all alarms should lead to false predictions. Figure 2 demonstrates that the PLV values between 0.6 and 0.85, which corresponds to the calculated PLV values. These higher PLV values correspond to channel synchronization (0.83–0.85). Figures 3, 4 correspond to PLV and ALV values, respectively, over interictal behavior. These values do not reach the patient specific threshold level of “0.83”, which demarcates non-seizure behavior from seizure behavior. Figures 5, 6 demonstrate the calculated PLV/ALV values over a time series featuring seizure activity. The calculated PLV for this data set shows a pre-ictal marker that rises above a selected threshold in Figure 5, which is denoted as “P” for the prediction marker. Figure 5 also illustrates the PLV rising above a threshold value that corresponds to a seizure event, denoted by the marker “S”. Figure 6 displays the ALV as it signifies the seizure event and demarcates the inter-ictal time period.

### EEG Data Set

Data sets were gathered online from the “CHB-MIT Scalp EEG Database”, which consists of EEG recordings from pediatric subjects with intractable seizures from the Children’s Hospital Boston^1^, (Goldberger et al., 2000). All signals were sampled at 256 samples per second with 16-bit resolution, and collected using the International 10–20 system of EEG electrode positions. Pre-processing of EEG data consisted of finding the standard deviation (SD) of all channels against each channel, and sorting the results in descending order. In this manner, we can locate the area of the highest seizure occurrences or working channel. The channel with the highest SD is selected as the working channel. We take the channel with lowest SD as our comparison or reference channel. These two channels will be used as input to our algorithm. Those EEG channels that contained artifacts (electrode movement instead of seizure behavior) were discarded.

### Phase Locking Thresholds

In order to separate basal neurological activity from pre-ictal and ictal behavior, a threshold is set to classify instances of phase locking behavior associated to seizure activity. After calculating the phase component of the signal and calculating the phase locking value for a segment of the time series, we derive significance levels based on the calculated phases of the compared neural neighborhoods. A synchronization index [0–1] is applied in order to determine the level of synchronization between oscillating groups, where PLV values approaching one demonstrate a high degree of phase synchronization. The 95th percentile of the distribution of the synchronization indices serves as significance level. A demarcation between lower and higher synchronization indices determines seizure activity found in the EEG time series.

Phase locking threshold tuning is accomplished on testing one out of three datasets per patient and validation on the last two datasets. Most pre-ictal and ictal behavior of the signal have distinct PLV/ALV values that approach “1” (Figures 5, 6) vs. inter-ictal or non-seizure behavior (Figures 3, 4). A threshold is placed between these two states in order to demarcate between these states through analysis of PLV/ALV values on the testing dataset and validated through continuous data processing on the next two datasets per patient. Each patient dataset will have its own respective seizure vs. non-seizure threshold demarcation. The number of data points used for optimal PLV/ALV calculations, i.e., the value “n” which refers to the window size to sum PLV/ALV values is tested as well and validated throughout the datasets. In order to compensate for cross-patient differences in signal phases and amplitudes, we measured the standard deviation (SD) of a period of non-seizure EEG data for each subject, i.e., S_AAj_ and S_APj_ for SD of the AA and AP respectively, and used that value to scale the thresholds for each patient. Amplitude threshold (SA_threshold_) and phase threshold (SP_threshold_) values were chosen experimentally by analyzing and comparing the patterns of changes for different occurrences of seizures and artifacts and selecting the most appropriate value where:

$${\text{S}}_{\text{AAj}}<{\text{SA}}_{\text{threshold}}$$

$${\text{S}}_{\text{APj}}<{\text{SP}}_{\text{threshold}}$$

## Results and Discussion

**Figure 7:**
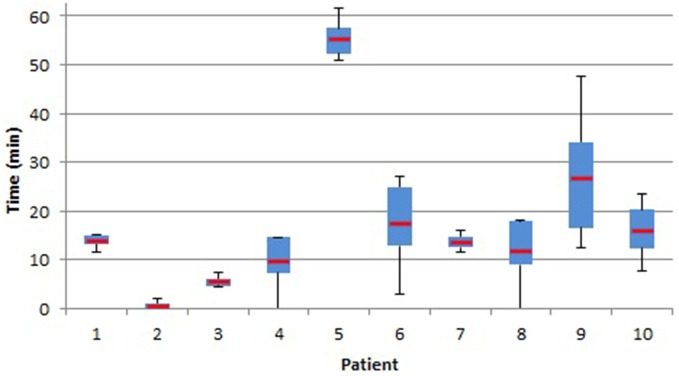
**Seizure prediction horizon (SPH; in minutes) of seizures predicted.** Box plot with whiskers display the distribution and median (red bar) of seizure activity for each patient.

EEG recordings were collected from ten patients during pre-surgical recording. EEG filtering was accomplished using a Remez filter in brain frequency ranges *delta-theta-alpha* (1–12 Hz), *alpha* (6−12 Hz), *beta* (13−30 Hz), *gamma* (30−40 Hz), and *upper-gamma* (40–50 Hz). SPHs ranged from 2 to 62 min, depending on band-pass filtering and patient, where patient “chb06” has the highest SPH among all the rest of the patients. The average SPH + SOP time period for *n* = 1000, 2500, and 5000 points is 20 min, across all band-pass ranges, as seen in Figure 7.

In order to determine the performance of our methodology, true positives are defined as those prediction markers that precede a seizure occurrence within 1 h, where false negatives are seizures that did not have bookmarked prediction. False positives are then defined as an SPH without the subsequent SOP event or an SOP event that existed outside the 1 h window. We have tested our patient-specific seizure prediction and detection algorithm for seizure prediction on 10 patients with 30 seizure events and 31 h of interictal recordings in the MIT-CHB EEG database. To evaluate the algorithm we have measured sensitivity (TP/(TP + FN)), precision (TP/(TP + FP)), the false alarm rate per hour, and the percentage of interictal recordings incorrectly classified as FPs. The false alarm rate per hour and the percentage of interictal data that is incorrectly classified as FPs demonstrate how many false alarms the proposed algorithm would generate.

Results for all the patients and all the four preprocessing methods are shown in Table 1. Each patient number has the associated MIT-CHB patient number. The gamma band-pass filter produced the highest prediction sensitivity and precision as well as the lowest FP occurrences, with total sensitivity of 77% (classifies 24 preictal events correctly out of 31), a total precision of 88% and 0.17 false positives per hour (three false alarm events in 31 h of interictal recordings). Due to the low occurrences of FPs, the precision rate produced higher results than the sensitivity rate.

**Table 1:** **Results from seizure prediction analysis by the proposed algorithm**.

Pat. No.	No. of Sz	Interictal hours	Sensitivity (%)	Precision (%)	FP/h	FP %	*p*-value
1 (1)	3	3	67	100	0.00	0.00	0.002
2 (2)	3	3	33	33	1.11	66.67	0.031
3 (3)	3	3	100	100	0.00	0.00	0.000
4 (5)	3	3	67	100	0.00	0.00	0.000
5 (6)	3	4	67	100	0.00	0.00	0.000
6 (11)	3	3	67	100	0.00	0.00	0.092
7 (18)	3	3	100	100	0.00	0.00	0.000
8 (20)	3	3	67	50	0.56	33.33	0.211
9 (22)	3	3	100	100	0.00	0.00	0.000
10 (24)	3	3	100	100	0.00	0.00	0.000

In order to test whether our algorithm is different from chance, we implemented a randomizing chance predictor using a Poisson process, whereby the probability of generating a preictal classification is uniformly distributed. According to Snyder et al. (2008), a period τ_w0_ is provided to accommodate uncertainty regarding the precise moment of seizure onset, and to distinguish seizure detection from seizure prediction. This parameter, τ_w0_, is referred to as the detection interval, while τ_w_ corresponds to the sum of SPH and the SOP of the earlier work. The proportion of time that the chance predictor spends in warning is ρ_w_. The calculation for the Poisson rate for the chance predictor is:

$$\lambda_{w}=\frac{1}{\tau_{w}}\ln(1-\rho_{w})$$

Snyder states that the probability of raising a preictal flag in a short interval of duration Δt is approximately equal to λ_w_Δt, independent of t, where λ_w_ is referred to as the Poisson rate parameter. We can calculate the sensitivity of the chance predictor:

$$S_{nc}=1-\exp\left(-\lambda_{w}\tau_{w}+(1-e^{-\lambda_{w}\tau_{w0}})\right)$$

To assess the significance of an improvement over chance, a candidate algorithm identifies *n* of *N* seizures (i.e., observed sensitivity *S_n_* = *n/N*) for an individual patient. The two-sided *p*-value is the probability of observing a difference *|n/N - S_nc_|* or greater if the algorithm under evaluation is not different from chance. This is shown to be equal to

$$p=\{\begin{array}{cc} \left[1-F_{\text{B}}(n-1;N,S_{nc})\right]+F_{\text{B}}(k_{f};N,S_{nc}), & \text{for}\frac{n}{N}\geq S_{nc}\\ \left[1-F_{\text{B}}(k_{\text{c}}-1;N,S_{nc})\right]+F_{\text{B}}(n;N,S_{nc}), & \text{for}\frac{n}{N}<S_{nc} \end{array}$$

where *k_f_* = ⌊2*NS_nc_* − n ⌋ and *k_c_* = ⌈2*NS_nc_* − *n*⌉. When algorithms are designed and applied prospectively to a population of patients, the values of ρ_w_ will vary from patient to patient. In this case, the overall significance involves the statistics of, e.g., the median sensitivity improvement, with corresponding hypotheses

$$\begin{array}{cc} {\text{H}}_{\text{0}}:\text{median}\;(\left(S_{\text{n}}-S_{nc}\right) & \text{for}\;\text{algorithm})\text{=0}\\ {\text{H}}_{1}:\text{median}\;(\left(S_{\text{n}}-S_{nc}\right) & \text{for}\;\text{algorithm})\neq \text{0} \end{array}$$

Prediction rates using band-pass filtering in the gamma range are significantly better than chance in 8/10 patients at a significance cutoff of *a* = 0.05 (Daniel, 2009; see Table 1).

Two of our proposed preprocessing methods significantly enhanced the prediction rate: window size and band-pass filtering. Window sizes between *n* = 1000, 2500 and 5000 points had a discernable impact on the total sensitivity and precision of the prediction methodology, yet a window size of *n* = 2500 reduced the sensivity to 64% (classifies 20 preictal events correctly out of 31), a total precision of 86%, demonstrating the significance of window size selection. The effects of band-pass filtering on prediction rates were more salient where sensivity and precision ranged from 54% (classifies 17 preictal events correctly out of 31) and 64% in the alpha range, 54–62% in the beta range and 64–79% in the low gamma range.

Band-pass filtering and window size selection for the algorithm led to a significant improvement in the false positive, such as the decrease in false alarm events to 3 from 10 in 31 interictal hours. If we reduce the SPH from 1 h to 30 min, since the average SPH is 20 min across all the patients, the total sensitivity would remain 77%, but total precision would reduce to 78% with 0.28 false positives per hour due to patient 5’s prediction values being classified as FP from TP.

Previous implementations utilizing the CHB MIT data base, utilized machine learning based classification of bivariate patterns method achieved 52.2% sensitivity (Chiang et al., 2011). Shoeb and Guttag (2010) utilized a SVM to construct patient-specific classifiers that use scalp EEG signals to detect the onset of epileptic seizures with a sensivity of 96%, but their approach detected seizures <10 s before their occurrence. The “dynamical similarity index” introduced by Le Van Quyen et al. (1999) compares the dynamic of the EEG data in a sliding window with the data in a fixed reference window of an interictal period. For SOPs up to 30 min, and a small SPH of 5 s, its sensitivity ranges from 21 to 42% (Winterhalder et al., 2003).

The results from Table 1 demonstrate that PLV/ALV analysis has a higher rate of detection of seizure vs. random chance detection. PLV/ALV detection can decipher random occurrences of phase locking events thereby detecting true neurological attributes found in seizures events.

The experimental findings illustrate that there may be a triggering mechanism through the PLV method that may enable better control of an implanted seizure control system through seizure prediction. In this manner, a pulse generator could be programmed to deliver electrical stimulation during the SPH time interval to the brain if the PLV value rises above a threshold range.

## Conclusion

The seizure prediction and detection methodology was tested on interictal and ictal events against a commonly available large data set, exhibiting a high degree of sensitivity and precision. It was observed through human EEG testing that the signal captured through high-density electrodes exhibited the following traits: (1) pre-ictal PLV values reach a threshold several minutes before the seizure event. This state may represent the initial imbalance of the electrical activity of the brain through seizure neuron firing. The phase of signal emanated from these neurons will be much longer than normal neuron firings. The SPH is defined as the SPH + SOP where the SPH initiates the seizure activity, followed by; (2) a reconstitution state where the brain attempts to restore the electrical activity in the presence of initial abnormal neuron firings. This effect is shown as PLV and ALV values return to below threshold values; and (3) the PLV/ALV values once again rise to the threshold value, signaling the seizure event and the last part of the SPH + SOP period. In this manner, the electrical activity of the brain is overcome by the abnormal neuron signals, and will eventually fall into an imbalanced state. This event is represented by the SOP part of the seizure activity. Through early seizure detection, this methodology can be implemented into a seizure control system that can aid in the management of recurrent seizure activity.

## Author Contributions

MHM developed the algorithm, literature review, statistical analysis and developed the manuscript. AP rigorously tested the algorithm against the online database. GH, ALdJC, and CDB provided additional analysis and editing of the manuscript.

## Conflict of Interest Statement

The authors declare that the research was conducted in the absence of any commercial or financial relationships that could be construed as a potential conflict of interest.

## Footnotes

^1^http://physionet.org/pn6/chbmit/
